# Hurler Syndrome (Mucopolysaccharidosis Type 1): A Case Report

**DOI:** 10.7759/cureus.37785

**Published:** 2023-04-18

**Authors:** Noman Khalid, Muhammad Abdullah, Abeer bin Awais, Muhammad Hassan, Ameer Muhammad

**Affiliations:** 1 Internal Medicine, Shaikh Khalifa Bin Zayed Al-Nahyan Medical & Dental College, Shaikh Zayed Medical Complex, Lahore, PAK; 2 Public Health & Community Medicine, Shaikh Khalifa Bin Zayed Al-Nahyan Medical & Dental College, Shaikh Zayed Medical Complex, Lahore, PAK; 3 Medicine, Shaikh Khalifa Bin Zayed Al-Nahyan Medical & Dental College, Shaikh Zayed Medical Complex, Lahore, PAK; 4 Internal Medicine, Shaikh Zayed Hospital, Shaikh Zayed Medical Complex, Lahore, PAK

**Keywords:** hurler syndrome, mucopolysaccharidosis type 1, lysosomal storage disorder, alpha l iduronidase, glycosaminoglycans

## Abstract

Hurler syndrome is a rare autosomal recessive disorder of deficiency in the metabolism of glycosaminoglycans (GAGs), including heparan sulfate and dermatan sulfate, which consequently accumulate in the different organs of the body, resulting from deficiency of an enzyme named Alpha-L-iduronidase. Here, we present an interesting case of a young female patient who presented with a combination of skeletal, oro-facial, ophthalmologic, neurological, and radiological findings of this disease. A diagnosis of Hurler syndrome (Mucopolysaccharidosis Type I) was made late in the disease due to lack of facilities, and the patient was ultimately managed supportively.

## Introduction

Mucopolysaccharidoses type I (MPS I) are a group of lysosomal storage disorders caused by a deficiency of alpha-L-iduronidase, an enzyme involved in the breakdown of glycosaminoglycans (GAG) or mucopolysaccharides [1,2]. Children affected are normal at birth, but deficient catabolism leads to progressive accumulation of heparan sulfate and dermatan sulfate in the tissues, resulting in the disease affecting bones, joints, eyes, heart, respiratory system, and neurocognition [1,3].

As MPS I is a disease caused by progressive accumulation of GAG, early treatment is of great importance to prevent irreversible pathologies and substantially improve a patient’s life expectancy [4-9]. Non-specific symptoms, variable presentations, and lack of disease awareness are some factors that prevent early and accurate diagnosis of the syndrome [10]. Here we present a case of Hurler syndrome that was diagnosed later in the course of the disease.

## Case presentation

A 10-year-old female presented to a pediatric outpatient setting with complaints of fever, cough, and noisy breathing. She had been followed regularly by the pediatric department, where her treatment was carried out since her diagnosis of Hurler syndrome at the age of six years. 

Her medical history revealed that she was born to second-degree consanguineous parents, but all family members, including her parents, are well. She was an average-size full-term neonate, and there were no complications at birth. She presented with bloating, vomiting, and on-and-off episodes of diarrhea at 18 months of age. These symptoms persisted for a long time despite symptomatic treatment. At the age of four, she had her first of many respiratory tract infections, i.e., fever, cough, and noisy breathing. The patient was brought to the hospital to consult her speech delay and on-and-off ear infections when she was 4.5 years of age. Later, she reported difficulty in nasal breathing and corneal clouding at eight years and 10 years, respectively. 

Gross facial dysmorphism was noted in the form of frontal bossing, broad-based nose, large protruded tongue, puffy eyes and face, and coarse facial features. She had a short neck, small chest, distended abdomen, umbilical hernia, clubbing of fingers, and flexion of the distal first interphalangeal joint. Her skin was pale, coarse, dry, and scaly with a dusky complexion. She weighed 9 kg, and her height was 91 cm, which showed her weight:height, height:age, and weight:age ratios were below the normal range. Abdominal examination revealed hepatosplenomegaly, and there was no shifting dullness or fluid thrill. Further, bilateral corneal opacities were found. 

Hematological investigations showed microcytic anemia along with a mild increase in alanine transaminase (ALT), aspartate transaminase (AST), and alkaline phosphatase (ALP). Urine tests (random/spot) showed elevated mucopolysaccharides. Enzyme analysis of alpha-L-iduronidase showed deficient enzyme levels, i.e., 0.3 umol/L of blood/h (normal range: 3.5-13 umol/L of blood/h). The thyroid hormone profile was normal. 

**Figure 1:**
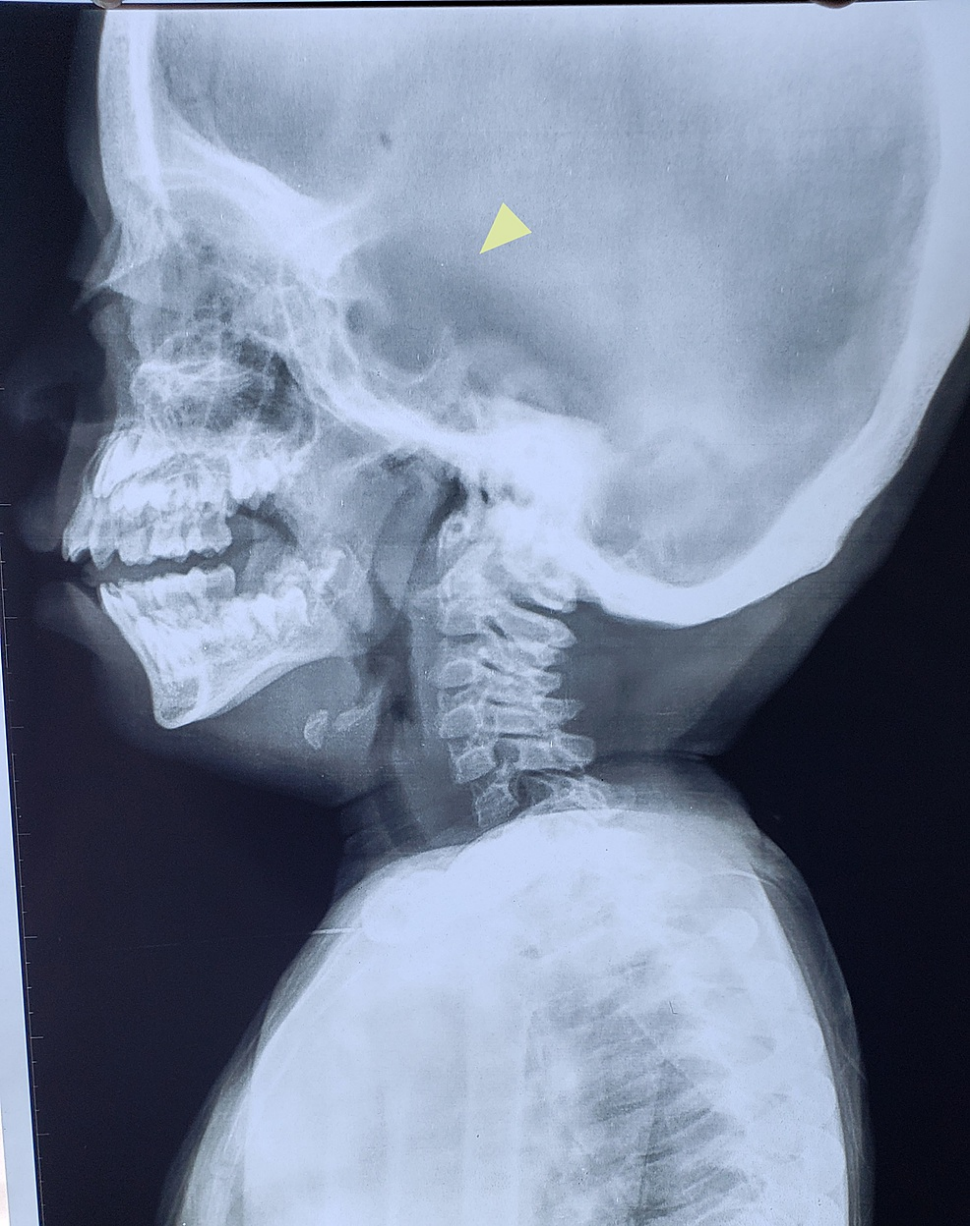
J-shaped sella

**Figure 2:**
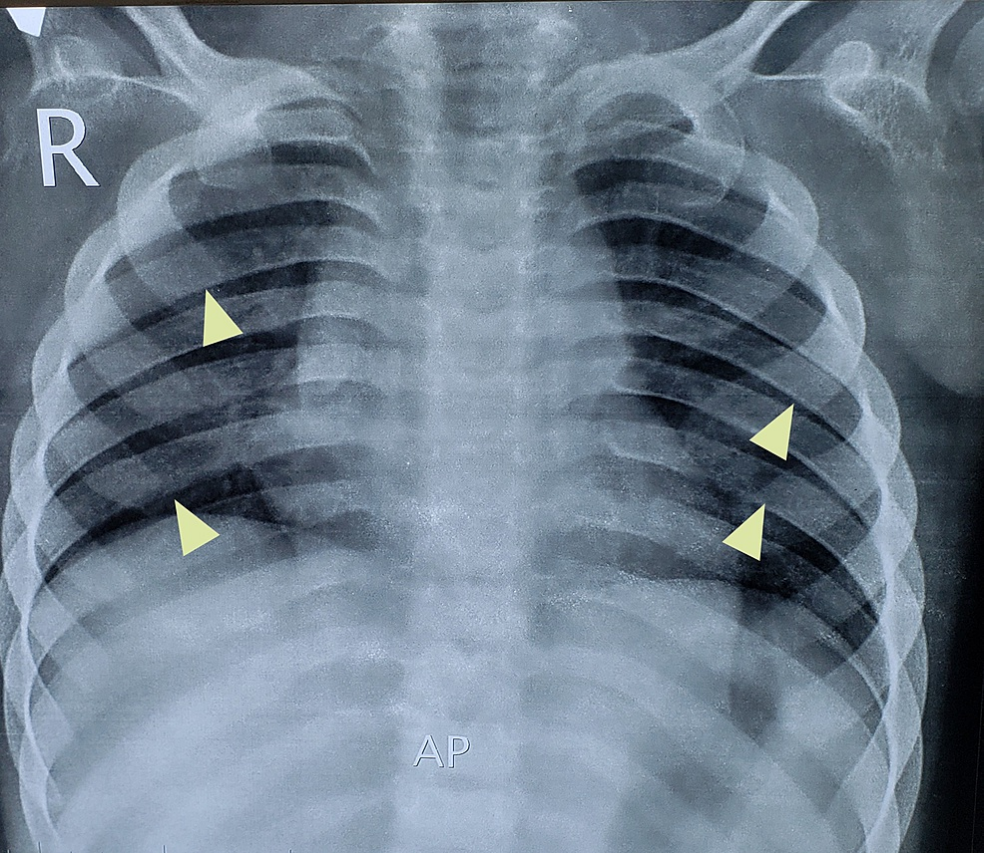
Oar-shaped/paddle ribs

**Figure 3:**
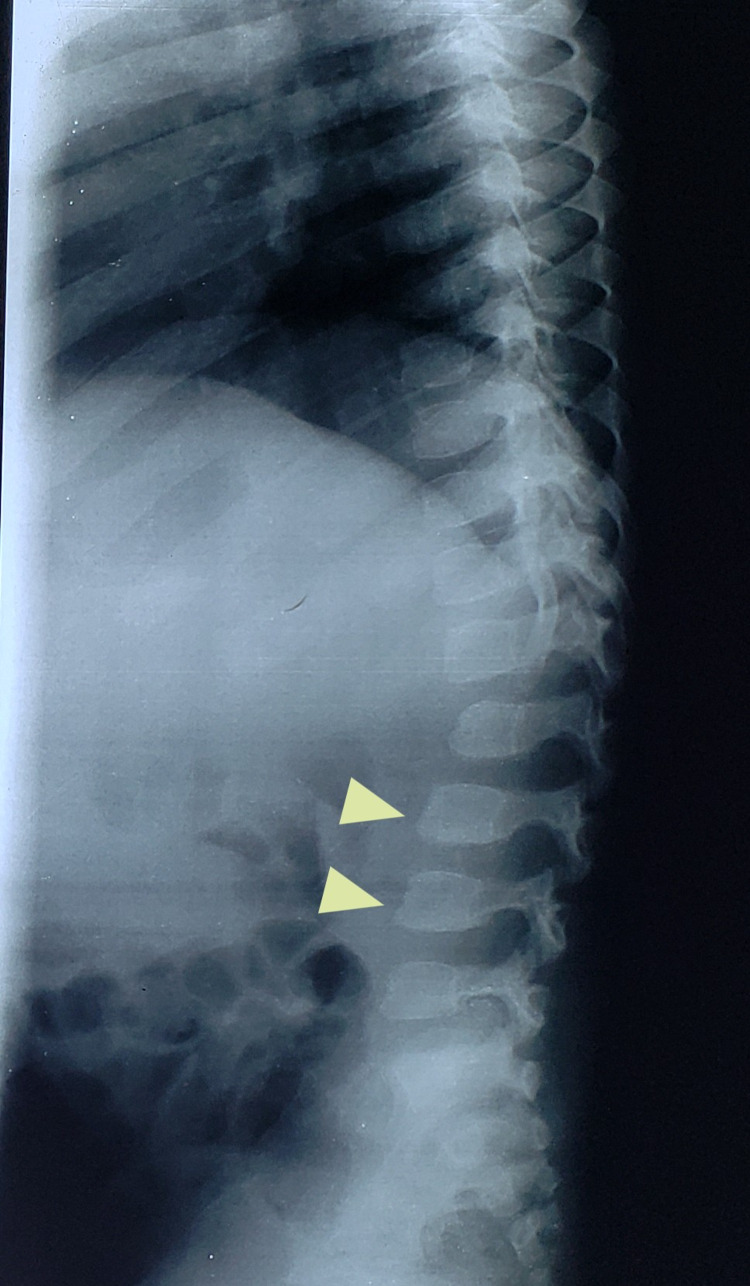
Anterior inferior vertebral body beaking

**Figure 4:**
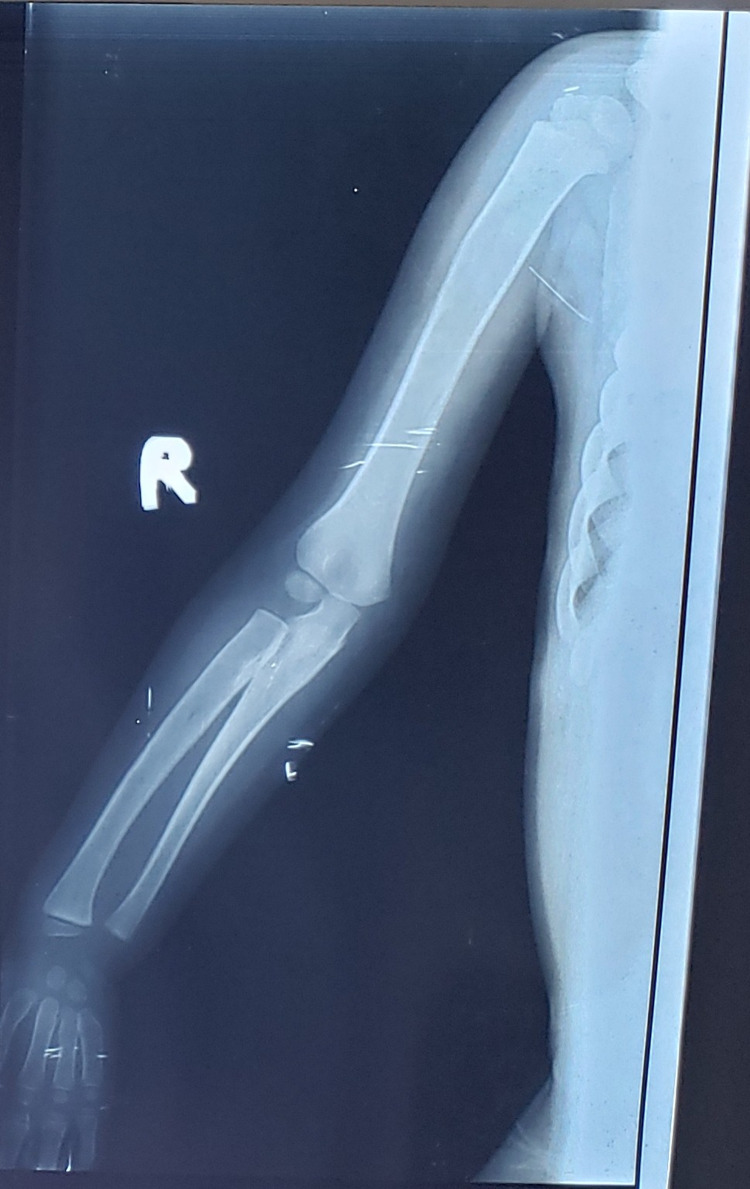
Radial and ulnar deformities

On radiological examinations, X-ray of the skull showed a J-shaped sella (Figure 1). On chest x-ray, flattened, oar-shaped ribs (narrowed at vertebral ends and wide at sternal ends) were seen (Figure 2). X-ray of the spine showed prominent inferior beaking (Figure 3). In an x-ray of the forearm and hands, there was angulation of the lower ends of the radius and ulna with slopping of lower surfaces towards each other, characteristic proximal tapering of metacarpal bones (Figure 4).

Pure tone audiometry findings showed a bilateral moderately severe degree of conductive hearing loss and a moderate degree of sensorineural hearing loss.

Management

Treatment was only done on a supportive basis, with artificial tear eye drops for corneal clouding, third-generation cephalosporins, macrolides for respiratory infections, and multivitamin supplements. Furthermore, hearing aids were advised for the hearing difficulty.

## Discussion

MPS I is further divided based on its severity, with Hurler syndrome (MPS I-H) being the most severe, followed by Hurler-Scheie (MPS I-H/S), and then Scheie syndrome (MPS I-S) being the mildest [11]. These three forms have many overlapping symptoms and cannot be differentiated from the enzyme assays [12]. The diagnosis is based on a deficient enzyme assay, and all forms have deficiency so that the distinguishing features may be the course of the disease [13].

As it is an inherited autosomal recessive disorder, the risk increases with an increasing degree of consanguinity [14]. Furthermore, it is also demonstrated in this case as the parents of this patient are second-degree cousins.

Hurler syndrome (MPS I-H), the most common and severe type, has an incidence of 0.76 in 100,000 live births with a median life expectancy of 8.7 years [15]. It has several characteristic features: the coarse facial profile, hepatosplenomegaly, hearing loss, joint stiffness, contractures, developmental delay, recurrent respiratory infections, dysostosis multiplex, corneal clouding, communicating hydrocephalus, and hearing loss [1]. Most of these features are present in this patient. Children with Hurler syndrome are normal at birth as seen in this patient but progressively begin to show manifestations of the disease due to the accumulation of GAGs and are diagnosed around six months to two years of age [16]. Diagnosis is based on clinical features, urinary GAG analysis, and enzyme activity assays [10]. The confirmatory test for diagnosis of MPS I is an enzyme assay demonstrating deficiency of alpha iduronidase [17].

Our patient presented first at the age of 1.5 years with abdominal distention as the presenting complaint and on-and-off diarrhea, and after rigorous workup and follow-up, at the age of 3.5 years, a diagnosis of MPS I was made by the radiologist after some characteristic findings on X-ray. The confirmatory enzymatic assay of alpha-L-iduronidase was done at the age of seven years. However, the confirmatory test was delayed because of the lack of testing facilities in the native country and the financial burden of sending the samples abroad.

Management includes enzyme replacement therapy with recombinant alpha-L-iduronidase, hematopoietic stem cell transplant, and other supportive measures [18,19]. Nevertheless, the lack of availability of therapeutic measures in the country and late diagnosis led to mostly supportive management for the patient.

## Conclusions

Diagnosis and treatment of Hurler syndrome in the developing world still lack in the 21st century; diagnosis is delayed, and treatment is scarce. This report shows a mismanaged case of Hurler syndrome, which was diagnosed late, and no specific treatment except supportive care was given due to the lack of availability of facilities. Clinicians should be able to reach a diagnosis and treat it appropriately earlier in the course of the disease to prevent the reoccurrences of this case and its potential harms. Together with a collaboration of funding and worldwide support, we can overcome the mismanagement and, along with that, improve the quality of life of Hurler syndrome patients. 
